# Chemical and Biological Sensors for Food-Quality Monitoring and Smart Packaging

**DOI:** 10.3390/foods7100168

**Published:** 2018-10-16

**Authors:** Fatima Mustafa, Silvana Andreescu

**Affiliations:** Department of Chemistry and Biomolecular Science, Clarkson University, Potsdam, NY 13699, USA; mustaffm@clarkson.edu

**Keywords:** sensors, smart packaging, food freshness, food quality

## Abstract

The growing interest in food quality and safety requires the development of sensitive and reliable methods of analysis as well as technology for freshness preservation and food quality. This review describes the status of chemical and biological sensors for food monitoring and smart packaging. Sensing designs and their analytical features for measuring freshness markers, allergens, pathogens, adulterants and toxicants are discussed with example of applications. Their potential implementation in smart packaging could facilitate food-status monitoring, reduce food waste, extend shelf-life, and improve overall food quality. However, most sensors are still in the development stage and need significant work before implementation in real-world applications. Issues like sensitivity, selectivity, robustness, and safety of the sensing materials due to potential contact or migration in food need to be established. The current development status of these technologies, along with a discussion of the challenges and opportunities for future research, are discussed.

## 1. Introduction

The growing food industry and increased demand for the long-term storage and preservation of food have created the need to develop methods that can easily track and preserve food freshness and safety throughout the shelf life of the product (production, storage, shipment, and consumption). Smart sensors and labels that can be attached to packaging represent next-generation technology that can help monitor the status of the product. These can be designed to measure markers of freshness and provide an “index of quality” of the product in real time, measure temperature changes, or identify the presence of harmful components. Additional capabilities can be added to provide protective functions, for example, packaging with coating that can act as an oxygen barrier to prevent spoilage [1]. These methods can replace costly techniques that are currently being used to monitor food products and increase sample throughput. Here, we describe the status of chemical and biological sensors for monitoring food quality and safety that can be used to improve shelf life and measurement capabilities of food products.

Traditionally, sensors are composed of chemical or biological receptors that are specifically designed to recognize a target analyte, and a physical transducer that converts the recognition process into a measurable signal, generating a quantitative and/or qualitative output [2]. Biological sensors or biosensors rely on the recognition properties of biomolecules such as enzymes, aptamers, and antibodies [3], which are integrated with the physical transducer to enhance selectivity for the target analyte. The binding process is monitored through a variety of methods, including colorimetric [4], electrochemical [5], optical [6], and mass-based detection [7]. While biosensors have greatly influenced the medical-diagnosis field, e.g., the widely used electrochemical biosensor for glucose monitoring, which accounts for 85% of the biosensors in the market [8], the use of biosensors in other fields such as food industry is still in its infancy, with few examples available.

Sensors have been described for raw-materials testing, authenticity evaluation, and identification of genetically modified materials [7], allergens [9], and pathogenic [10] or chemical contamination. Incorporation of sensors in smart packaging is increasing [11] and has found some commercial success for food-freshness assessment [12], with notable examples for chemical rather than biological sensors. This review provides an overview of the development and implementation of chemical and biological sensors used for food-monitoring applications and their potential use as labels in smart packaging. Some commercial examples are discussed along with emerging technologies from the scientific research. Moreover, future trends in research and development of smart packaging are discussed.

## 2. Current Status of Active and Functional Packaging

**Figure 1:**
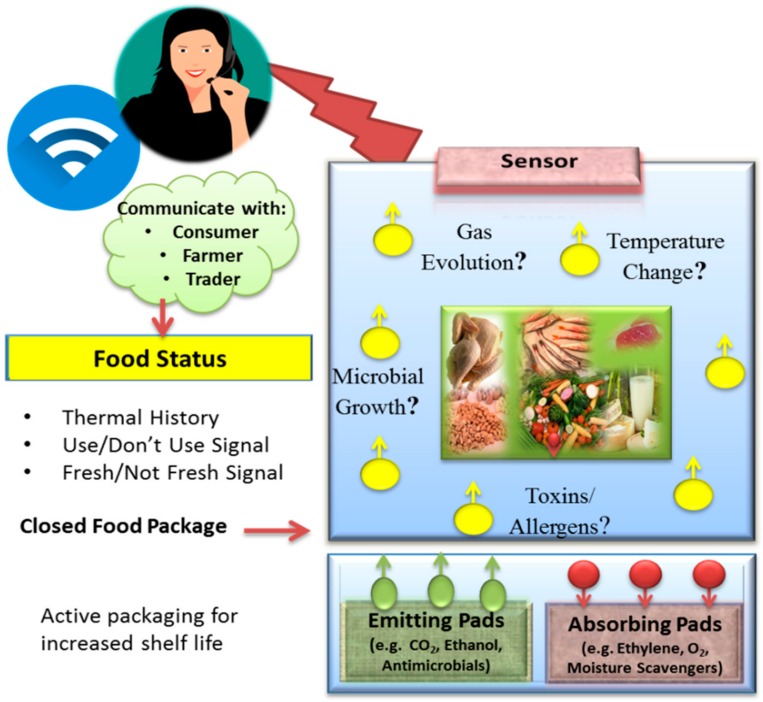
Representation of the concept of smart and active packaging technology.

Conventional packaging methods have been used not only to facilitate product handling, but also to preserve nutrition value, extend their shelf life, and reduce spoilage. Recent efforts are designed to develop smart and active packaging technologies [13] that are able to provide additional functions, such as detection and communication to inform consumers when spoilage occurs, in addition to preserving the product [1]. Examples of active packaging include the use of absorbing or emitting sachets/pads [14], as shown in Figure 1. Absorbing sachets could contain O_2_ scavengers [15,16] to decrease fat oxidation, ethylene scavengers to minimize fruit and vegetable ripening [17,18,19], humidity absorbers, odor absorbers, and antimicrobial growth inhibitors [20]. Emitting pads include CO_2_ emitters inhibiting microbial growth in meat [21], antimicrobial preservative releasers to minimize spoilage due to bacterial growth [22], and antioxidant releasers to reduce oil and fat oxidation [23].

Smart packaging can incorporate sensing platforms that provide information about the quality of food through the entire food chain such as composition, storage conditions, and bacterial growth [24]. The concept is depicted in Figure 1. Existing systems include time temperature indicators (TTI), which provide a thermal history of the product during time of storage, and distribution, enabling the consumer or the manufacturer to assess the product status [25]. For instance, MonitorMark^™^ a TTI sensor developed by 3M^™^ (3M^™^, Maplewood, MN, USA) was designed to monitor thermal exposure for meat, fish, and dairy products during storage and transportation below 20 °C [26]. Another example is the CoolVu indicator [27] developed in Freshpoint-Switzerland, which includes a metal and a transparent label. The label is composed of an etchant material and it is designed to provide a visual “use”/“do not use” change to inform customers of product-quality changes.

**Figure 2:**
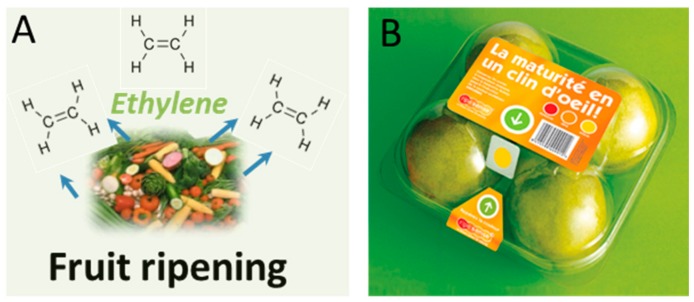
(**A**) Schematic illustrating the release of ethylene during fruit ripening; (**B**) example of a ripening label by RipeSense^®^ (RipeSense, Auckland, New Zealand) placed on the top of the package, where color changes from red to yellow according to the ripeness degree. Color development originates from the reaction of evolved gases with the label. Reproduced with permission from Reference [31].

Other platforms have been developed to respond to the generation of gas during ripening processes in the packaging headspace [28]. Some systems enable remote monitoring via radio-frequency identification (RFID) [29]. An RFID sensor for the detection of *Escherichia coli* (*E. coli*) and *Salmonella* in packaged foods was designed by Flex Alert [30]. This technology is based on antitoxins immobilized on flexible RFID tags incorporated within packaging. Ideally, the system is connected to a wireless network and produces a visual alert for real-time monitoring by farmers and producers. Another system, RipeSense^®^ (RipeSense, Auckland, New Zealand) [31] was designed as a smart ripeness-indicator label, developed in New Zealand. The system is able to communicate the ripeness degree of fruits without the need to open the package, but only by observing the change in the label color reacting with the gases evolved from the fruit, placed on the top of package. Among evolved gases, ethylene is the most widely used ripening indicator that is released during the ripening process [28] (Figure 2).

## 3. Food Freshness/Quality Monitoring

Several changes can take place in packaged food as a result of metabolism or microbial growth over time. For example, changes in gas evolution or microbial accumulation can be used to obtain information about the status of food, e.g., freshness or degradation [32]. Sensors that can measure such changes could provide an overall estimation of food quality. Examples include “on-package” pH indicators that change color when food decays as a result of pH changes associated with the release of volatile amines generated during meat or fish spoilage [33,34]. In this section, several developed and commercially available freshness indicators are described for different types of food including fish, meat, and poultry, cereal grains, fruits, and vegetables.

### 3.1. Fish, Meat, and Poultry

When meat, fish, or poultry undergo degradation, different spoilage indicators can be found indicating lipid decay, protein breakdown, and adenosine triphosphate (ATP) decay. The speed of degradation is dependent on the type of product, storage temperature, feeding habits, and harvesting methods. Traditional methods to assess freshness rely on human senses; although they are essential, they provide no quantitative data of spoiled food. Methods that can quantitatively measure markers of degradation through chemical or biological reactions can provide the means to more precisely assess the status and quality of food. In fish products, for example, one of the main freshness indicators is hypoxanthine, which is produced by the metabolic degradation of ATP [35]. Karube et al. (1984) [36] developed an equation for fish freshness assessment based on the content of inosine 5-phosphate, inosine, and hypoxanthine. Several enzymatic biosensors with colorimetric [37,38,39] or electrochemical detection have been developed to quantify the level of hypoxanthine [40] using the enzyme xanthine oxidase for biorecognition of hypoxanthine or xanthine [41,42]. An electrochemical biosensor prepared by immobilizing xanthine oxidase on a carbon-paste electrode modified with gold nanoparticles was reported and tested on chicken and meat samples, with a limit of detection of 2.2 × 10^−7^ M hypoxanthine [43]. To develop the sensor, xanthine oxidase (XOD) was immobilized by cross-linking with glutaraldehyde and bovine serum albumin (BSA) on different types of electrodes: carbon-paste electrodes and electrodeposited gold-over-gold disks. The highest sensitivity was obtained in the case of XOD immobilization on a carbon paste electrode modified with gold nanoparticles (AuNPs). The sensor was tested at operating potentials between 0.00 and 0.6 V and showed the possibility of working at 0.00 V, which enables the elimination of interfering compounds such as ascorbic acid. Detection of hypoxanthine by XOD involves the following steps in which hypoxanthine is first oxidized to xanthine, and then to uric acid:

$$\;\mathrm{Hypoxanthine}\;+{\;O}_{2}\overset{\mathrm{XOD}}{\to }\mathrm{Xanthine}\;+{\;H}_{2}O_{2}\;$$

$$\;\mathrm{Xanthine}\;+{\;O}_{2}\overset{\mathrm{XOD}}{\to }\mathrm{Uric}\;\mathrm{acid}\;+{\;H}_{2}O_{2}\;$$

**Figure 3:**
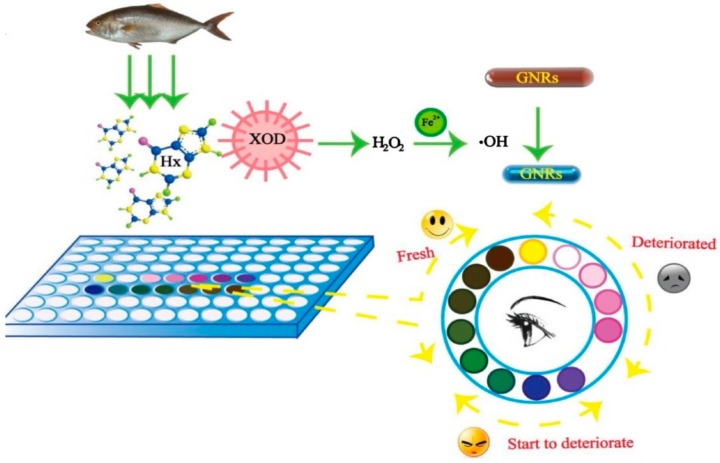
Example of colorimetric freshness sensor for fish via hypoxanthine detection (reproduced with permission from Reference [38]).

Yan et al. (2017) [44] reported a colorimetric sensor for xanthine detection using a copper nanocluster with peroxidase-like activity. The use of copper nanoclusters showed to enhance the oxidation of 3,3′,5,5′-tetramethylbenzidine dye (TMB) in the presence of H_2_O_2_ formed from the oxidation of xanthine. The sensor exhibited a detection limit of 3.8 × 10^−7^ M, and a linear range from 5.0 × 10^−7^ to 1.0 × 10^−4^ M. Chen et al. (2017) [37] developed a multicolor sensor for hypoxanthine detection by using gold nanorods (GNRs). The H_2_O_2_ produced from hypoxanthine oxidation by XOD undergoes a Fenton reaction to produce hydroxyl radicals in the presence of Fe^2+^. In the presence of hydroxyl radicals, the GNRs are etched forming a vivid color change. Different colors were generated, such as reddish brown, gray, green, blue, purple, pink, and yellow, depending on the concentration of hypoxanthine in the range of 0–1.13 mM. The sensor showed the ability to semiquantitatively assess the levels of hypoxanthine in fish extract by the naked eye (Figure 3).

Other biomarkers for the identification of fish degradation are biogenic amines such as putrescine, cadaverine, histamine, tyramine, spermidine, spermine, and tryptamine that accumulate as a result of microbial decarboxylation of amino acids [45]. The most common measurement systems for the detection of biogenic amines are those based on the use of the diamineoxidase (DAO) enzyme, which catalyzes the oxidation of biogenic amines to their corresponding aldehyde, hydrogen peroxide, and ammonia. This can be detected by colorimetric or electrochemical means. Enzymatic biosensors for biogenic amines were demonstrated for analysis of cheese and anchovies degradation [46]. The DAO enzyme was immobilized on Pt or Au electrodes and entrapped between electrosynthesized polypyrrole (PPY) and poly-*ortho*-phenylenediamine (PPD) layers. The sensor showed high sensitivity, with a limit of detection (LOD) in the range of 6–12 μM of histamine, putrescine, and cadaverine and a stability of about three weeks with 87% retention of initial sensitivity. The sensor showed potential as a screening tool for the presence of biogenic amines.

Trimethylamine oxide is another marker that can be used to assess fish decomposition. Trimethylamine oxide degrades to trimethylamine increasing the fishy odor [47]. A colorimetric system for the detection of trimethylamine oxide has been developed by using pH indicator dyes immobilized on cellulose microparticles [48] that change color from green to red when the food is spoiled. The particles were embedded into food-grade silicone and were safely integrated into food packaging. The system showed no leaching, and cytotoxicity tests confirmed compatibility. Another example was described by using dyes loaded within porous TiO_2_ nanoparticles [49]. In other examples, total volatile basic nitrogen (TVB-N) was quantified by an organic semiconductor gas sensor with a porous top metal electrode sensor with a nanostructured surface for enhanced gas adsorption [50]. The sensor was able to detect 100 ppb where the accepted level for ammonia is 200–300 ppb. A colorimetric sensor to detect TVB-N such as ammonia and dimethylamine was fabricated with the pH-sensitive dye bromocresol green [51] and tested in fish packaging. Although the sensor exhibited a color change from yellow to blue when exposed to the evolved TVB-N from fish spoilage, a false-negative signal may occur. Quantification of volatile organic compounds has also been used to evaluate meat, fish, and poultry freshness, for example, the system used to build the Swiss sensor FOOD sniffer [52]. Distell developed a Fish Freshness Meter and Torrymeter to measure fish freshness [53]. The sensing system is based on measuring the dielectric properties of fish-flesh skin when it starts to spoil, providing a numerical read out that reflects the degree of freshness. Portable meat fish fat meters based on water-content measurements, which is proportional to fat content, were also developed and are available.

Microbial activity in meat leads to the generation of compounds such as NH_3_, CO_2_, and H_2_S due to decarboxylation, deamination, and desulfurization of amino acids. Visual freshness monitoring of skinless chicken breasts was performed by detecting CO_2_ metabolites with mixtures of pH-sensitive dyes (bromothymol blue, bromophenol blue, bromocresol purple, methyl red, bromocresol green, methyl orange, methyl yellow, phenol red) [54]. The most sensitive mixture of dyes was composed of bromothymol blue and methyl red, which turned from green to yellow when the meat starts to deteriorate. Although several studies have been reported in the literature to develop sensors for meat and fish packaging, there is a general lack of toxicity investigations and the long-term effects of the sensing materials proposed for food packaging.

### 3.2. Cereal Grains

**Figure 4:**
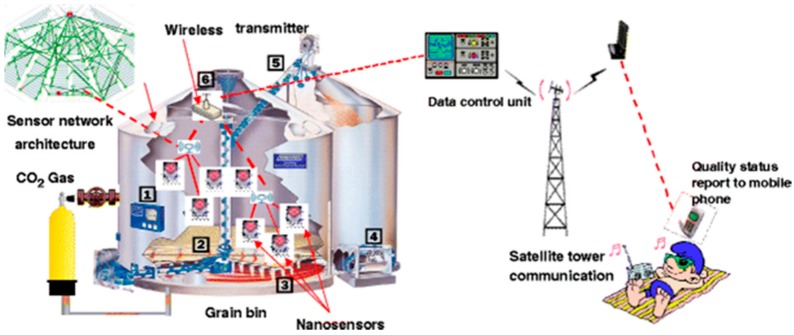
Futuristic nanosensor based on wireless network for grain-spoilage detection. (**1**) Control panel, (**2**) grain auger, (**3**) air plenum, (**4**) fan, (**5**) auger to transfer grain, if needed, (**6**) wireless transmitter (reproduced with permission from Reference [1]).

One of the indicators of grain spoilage during storage is the emission of CO_2_ as a result of insect infestations, and mold spoilage [55] causing grain deterioration or the production of harmful mycotoxins [56,57,58]. Developing CO_2_ sensors for early spoilage detection has been reported [59,60]. Neethirajan et al. (2010) [61] developed a sensor based on polyaniline boronic acid (PABA) conducting polymer for measuring CO_2_ levels in the range of 380–2400 ppm in simulated grain bulk. The sensing mechanism is based on the conversion between the emeraldine salt form and the insulating emeraldine base form of polyaniline and PABA through protonation and deprotonation. When gaseous CO_2_ reacts with water, it creates carbonic acid that protonates the polyaniline and further increases conductivity as CO_2_ partial pressure increases. However, the sensor performance was found to be dependent on the relative humidity, and further work should be done to enhance sensitivity and response time. An example of an advanced sensor concept with wireless transmission for large-scale monitoring of this type of spoilage, as well as the source in the grain bin, are shown in Figure 4 [1].

**Figure 5:**
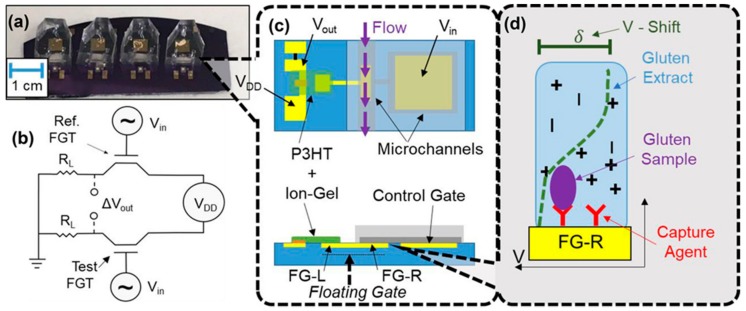
Floating-gate transistor (FGT)-based electronic biosensor structure. (**a**) Image of FGTs. (**b**) Circuit for a pair of FGTs. (**c**) Single FGT schematic showing electrodes, materials, and sample flow. (**d**) Sensor surface modified with antibodies or aptamers on floating-gate electrode (FG-R), upon binding to gluten, a change in the potential occurs (green line) leading to a shift in voltage value (reprinted with permission from Reference [65]. Copyright 2018, American Chemical Society).

Gluten is another component of interest for grain analysis, as certain individuals can develop gluten intolerance that can cause serious disorders of the digestive system [62]. The most common method for gluten analysis is by the conventional enzyme-linked immunosorbent assay (ELISA) [63]. Recently, an antibody-based device has been reported to analyze the gluten content in a variety of food samples in only 3 min [64]. Gliadin, a fraction of gluten was also detected using an impedimetric aptasensor with an LOD of 5 ppm, lower than the maximum allowed level for gluten-free products of 20 ppm [63]. The biosensor was tested for gluten content and gluten-free food, and was comparable with the ELISA technique. White et al. (2018) [65] developed an electronic sensor based on a floating-gate transistor (FGT). Both antibodies and aptamers were used as selective receptors for binding different types of gluten sources, such as wheat and barley. The sensor principle and its surface modification are shown in Figure 5. The antibody or aptamer-modified surface on floating-gate electrode (FG-R) is susceptible to potential change (green dotted line), producing a voltage shift.

Analysis of toxicity caused by mycotoxin contamination in cereals is also of special interest. Mycotoxins, such as ochratoxin A (OTA), aflatoxins, trichothecenes, fumonisins, zearalenone, and ergot alkaloids, are produced by fungi [66]. Several biosensor types for mycotoxin detection have been reported and reviewed extensively [56,67,68,69]. Electrochemical biosensors were developed for the detection of OTA using a competitive mechanism using OTA-specific aptamers and horseradish peroxidase (HRP) enzyme [70]. An OTA-specific aptamer was immobilized on paramagnetic microparticles, which compete with the HRP–OTA conjugate and free OTA in the developed assay. The beads were drop-casted over a screen-printed electrode, and the product of the enzymatic HRP reaction was detected using differential-pulse voltammetry. The sensor was applied for OTA detection in wheat in the linear range of 0.78–8.74 ng/mL and LOD of 0.07 ± 0.01 ng/mL. In another work, ceria nanoparticles functionalized with OTA-specific aptamers were used as enzyme-mimetic probes for colorimetric OTA detection [71]. OTA binding caused changes in ceria-particle reactivity, which was assessed by measuring TMB oxidation by ceria. The assay was able to detect down to 0.15 nM OTA. Overall, estimation of allergens and mycotoxins in cereals requires sample pretreatment and extraction of the targeted component; thus, direct application of these sensing mechanisms in food packaging is challenging.

### 3.3. Fruits and Vegetables

Fresh fruit and vegetable production is drastically increasing globally as a result of increased demand [72]. In the United States, about 40% of food is wasted, causing a yearly loss of about $165 billion [73]. Fruits and vegetables are highly perishable and can therefore easily degrade before reaching the consumer. Technology for monitoring and preserving fruits and vegetables is necessary to decrease food loss during transportation and storage [74]. Many fruits and vegetables produce ethylene due to environmental stress after being harvested. Ethylene can enhance ripening even at extremely low concentrations [75,76]. The presence of aging fruits and vegetables close to fresh ones can also cause aging and ripening as ethylene is emitted. Ethylene can be removed by using ethylene absorbers or oxidizers (scavengers). Scavenging systems facilitate removal, thus lowering the loss of other products due to overproduced ethylene. The most available ethylene scavenger is potassium permanganate (KMnO_4_), which oxides ethylene to ethylene glycol and can be further oxidized to CO_2_ and H_2_O, producing dark brown MnO_2_. Several commercial scavengers have been developed based on ethylene chemisorption by KMnO_4_ granules over clays or activated carbon [77]. Jiang et al. (2013) [78] suggested an ethylene-removal method by oxidation at low temperatures over a platinum catalyst on mesoporous silica. The catalyst was able to remove 50 ppm ethylene even at 0 °C.

**Figure 6:**
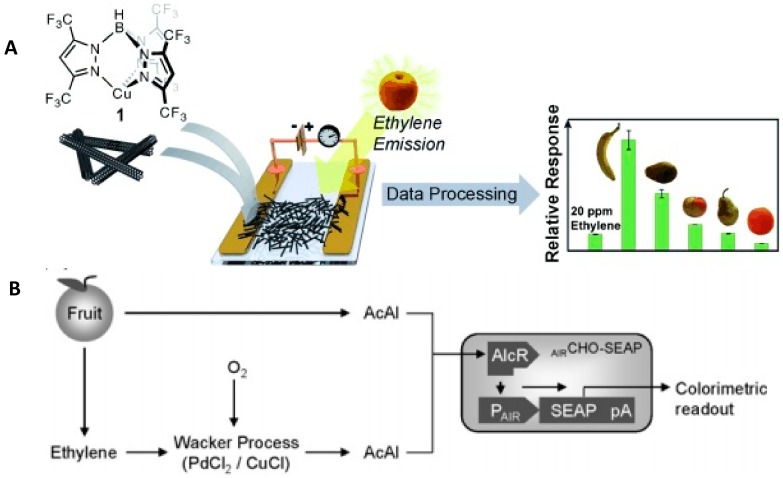
(**A**) Schematic representation of an ethylene chemoresistive sensor. Mixture of a Cu (I) complex and single-walled carbon nanotubes (SWNTs) were drop-cast between gold electrodes. When ethylene binds to the mixture, resistance changes. (Reproduced with permission from Reference [28]). (**B**) Dual-channel catalytic-biosensor. Acetaldehyde (AcAl) generated by fruit diffuses through the gas phase to biosensor cells (_AIR_CHO-SEAP) genetically engineered to express the *Aspergillus nidulans*-derived transactivator AlcR that, in the presence of acetaldehyde, activates its cognate promoter PAIR, driving expression of the reporter gene SEAP (AIRCHO-SEAP cells). Ethylene is oxidized to acetaldehyde on PdCl_2_ with Cu^+^ based on the Wacker process. The generated acetaldehyde is captured by _AIR_CHO-SEAP and converted into SEAP expression, measured as a colorimetric signal (reproduced with permission from Reference [78]).

To control produce freshness, ethylene sensors could be useful for detecting rapid ripening and prevent fruit degradation. Several sensors for ethylene detection have been reported. Esser et al. (2012) [28] developed a chemoresistive sensor made of single-walled carbon nanotubes (SWNTs) mixed with a Cu (I) complex placed between gold electrodes. Upon binding to ethylene, a resistance change occurred (Figure 6A); however, the sensor generated a significant signal of tested solvents such as acetonitrile and tetrahydrofuran, but the low concentrations of these compounds had little effect. Weber et al. (2009) [78] developed a biosensor for the detection of ethylene and acetaldehyde produced by plants. In their system, ethylene was oxidized to acetaldehyde on PdCl_2_ with Cu (I) based on the Wacker process, and the generated acetaldehyde was detected by a CHO-K1-derived sensor cell line _AIR_CHO-SEAP (Figure 6B).

**Figure 7:**
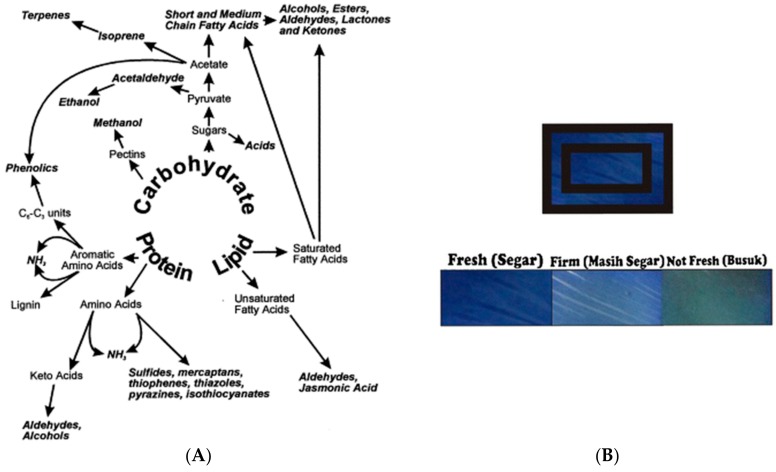
(**A**) Volatile organic compounds (VOC) that could accumulate in the presence of fruits and vegetables (reproduced with permission from Reference [79]); (**B**) VOC freshness indicator in guava packaging (reproduced with permission from Reference [80]).

Volatile organic compounds (VOC) accumulate in the presence of fruits and vegetables in closed containers or packages. Indicators for the detection of these compounds, such as terpenes, carboxylic acids, alcohols, aldehydes, sulfur compounds, ammonia, and jasmonates [79], have been reported (Figure 7A). A color-based pH indicator, developed using bromophenol blue immobilized on a cellulose membrane, enabled the detection of VOC (e.g., acetic acid) evolution in the headspace of guava packaging (Figure 7B) [80]. This label provides the consumer with the freshness status of guava; however, more investigations are needed to ensure the safety of using chemical dyes and limit the probability of migration into the food.

## 4. Biosensors in Food Analysis

The need for simple, rapid, and field-portable analytical methods has boosted development of biosensors for food analysis. The integration of biomolecules, such as enzymes, immunosystems, tissues, organelles, or whole cells, with a variety of transduction methods, such as electrical, thermal, or optical signals, has enabled the development of a wide array of biosensing devices [81]. Their selectivity and relative ease of analysis make them advantageous for use in food analysis. The development of biosensors in this field is described with biosensor examples for the detection of pathogens [82], allergens [83], and other toxicants, such as pesticides [84] and mycotoxins. In this section, we briefly highlight the application of biosensors for allergens, toxicants, and pathogen detection.

### 4.1. Biosensors for Food-Allergen Detection

**Figure 8:**
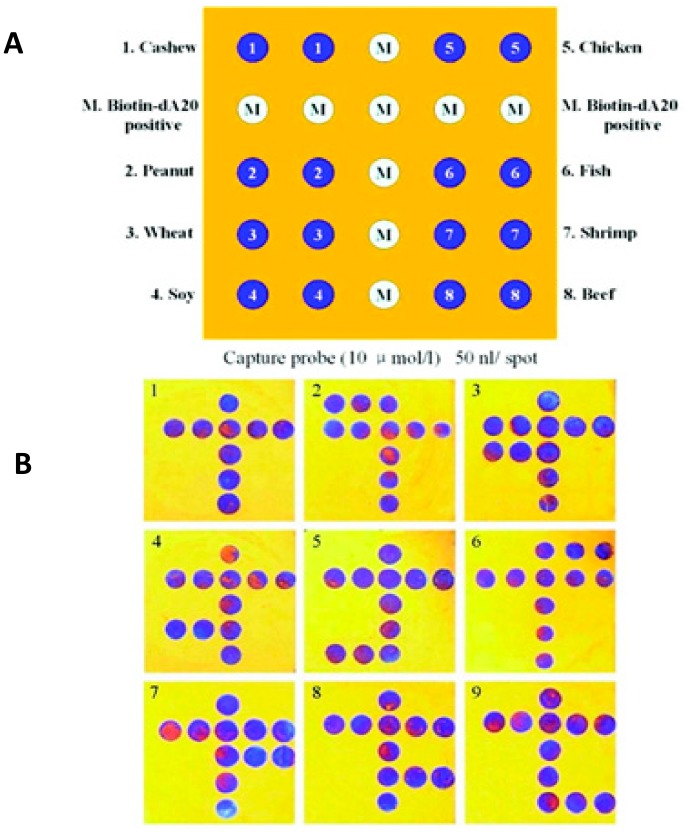
Allergen-detection chip. (**A**) Food probes were spotted in addition to positive control and marker (M); (**B**) detection of corresponding allergens: 1, H_2_O; 2, cashews; 3, peanuts; 4, wheat; 5, soybeans; 6, chicken; 7, fish; 8, shrimp; 9, beef (reprinted with permission from Reference [89]. Copyright 2011, American Chemical Society).

The presence of allergens in food products such as milk, soybeans, crustaceans, eggs, gluten-containing cereals, peanuts, and nuts (e.g., almonds, Brazil nuts, cashews, walnuts) is an increased safety concern, as prevalence of food allergies due to even trace amounts of allergens is increasing. About 10% of preschool children in industrial countries suffer from clinical food allergies [85]. A variety of DNA or immune-based biosensors have been developed for allergen detection [86], but in many cases sample preparation and purification are laborious and time-consuming [9]. Employing antibody-based detection and magnetite beads, NIMA Company (San Francisco, CA, USA) has developed a sensor to detect peanut allergens in ppm [87]. Archin (Ara h1) peanut allergens were detected by surface plasmon resonance (SPR)-immune-based biosensor in chocolate candy bars [88] with an LOD of 0.09 μg/mL for the optimized system. Three systems were tested and compared for the detection of the Ara h1 allergen: a label-free assay, a secondary antibody sandwich assay, and an NPs-based signal-enhanced SPR biosensor consisting of functionalized magnetic nanobeads. The SPR biosensor with NP enhancement provided the best sensitivity among the three assays, with a detection limit of 0.09 μg/mL, a linearity range from 0.1 to 2 μg/mL, and good correlation with a commercially available ELISA kit. Wang et al. (2011) [89] developed a colorimetric, silicon-based, optical thin-film biosensor chip with PCR amplification and demonstrated the ability to simultaneously identify eight food allergens found in soybeans, wheat, peanuts, cashews, shrimp, fish, beef, and chicken (Figure 8), with an LOD of 0.5 pg. The presence of gluten in food such as wheat, barley, and rye causes celiac disease for individuals who are unable to digest gluten [90]. A label-free electrochemical immunological sensor for β-lactoglobulin, an allergen usually found in milk, was developed with an LOD of 0.85 pg/mL [91]. The sensor was fabricated on a graphene-modified screen-printed electrode with immobilized β-lactoglobulin antibodies. Binding of β-lactoglobulin was quantified by measuring the reduction peak of [Fe(CN)_6_]^3−/4−^, which linearly decreased with the increase of β-lactoglobulin concentration. The sensor was applied to screen several samples such as cheese snacks, cake, and sweet biscuits, and the results were comparable with the commercial ELISA assay.

In another assay, allergy-causing proteins, such as casein and β-lactoglobulin (β-LG), in milk have also been detected based on aptamer recognition with the aptamer immobilized on a graphene-modified screen-printed electrode with voltammetric detection [92]. The method was tested on spiked milk samples and showed no significant matrix effect. Another common allergen in milk is casein. A casein immunosensor with Localized Surface Plasmon Resonance (LSPR) detection and immobilized casein antibodies was reported to detect casein in raw milk with an LOD of 10 ng/mL [93].

**Figure 9:**
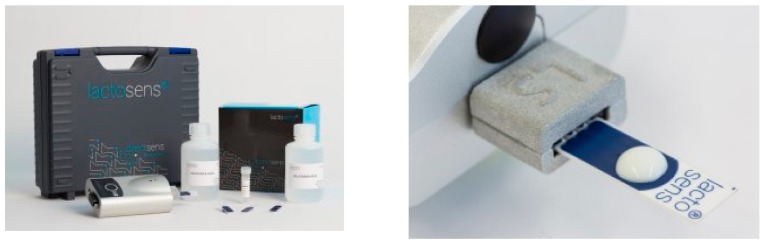
LactoSens^®^ enzymatic biosensor for lactose detection in milk. Reproduced with permission from DirectSens^®^ (Directsens, Klosterneuburg, Ausria) [100].

Lactose intolerance is found in all parts of the world forming about 15% of Northern Europeans, 80% of black people and Latinos, and about 100% American Indians and Asians [94]. It occurs in individuals lacking the enzyme (β-galactosidase) responsible for digesting lactose sugar into galactose and glucose [95]. Several types of electrochemical biosensors have been designed for lactose quantification utilizing coimmobilized β-galactosidase and glucose oxidase enzymes [96,97,98,99]. Several enzymatic lactose sensors are also available for the detection of trace amounts of lactose in lactose-free milk products, for example, LactoSens^®^ (Directsens, Klosterneuburg, Austria) biosensors. The enzymes are immobilized on disposable test strips, and lactose content is directly quantified by a reader (Figure 9) [100].

### 4.2. Biosensors for Bacterial-Pathogen Detection

**Figure 10:**
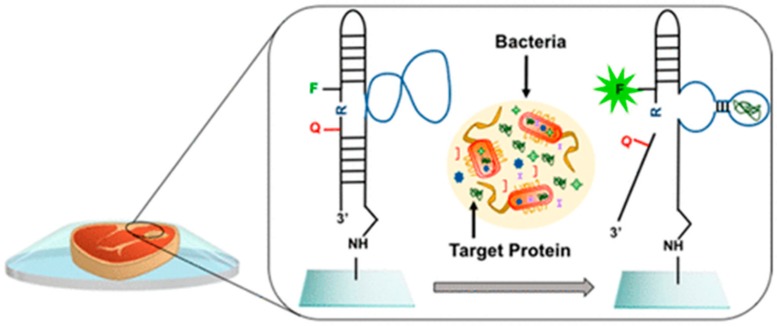
Fluorescent DNAzyme probe with specific binding characteristics for *E. coli* was printed on cyclo-olefin polymer transparent package (reprinted with permission from Reference [112]. Copyright 2018, American Chemical Society).

According to the Centers for Disease Control and Prevention (CDC) in the United States, the estimated number of people infected by bacteria-, virus-, and parasite-caused diseases is approximately 48 million [101]. The World Health Organization (WHO) Foodborne Disease Burden Epidemiology Reference Group (FERG) estimated 600 million foodborne illnesses with 420,000 deaths in 2010 globally [102]. The main causes of these diseases are pathogenic bacteria *Escherichia coli* and *Salmonella* causing most foodborne outbreaks in the United States [103]. Rapid detection of pathogenic bacteria plays an important role in food analysis. The main methods for pathogen detection are based on Polymerase Chain Reaction (PCR) or plate counting, which require sample enrichment and long analysis time [104]. Biosensors represent a possible alternative for pathogens detection due to their portability and potential for onsite detection. Most biosensors for bacterial-pathogen detection are those based on immune and DNA recognition, but these require extensive preparation procedures, involve labeling, multiple washing steps [105,106], and specialized facilities [107]. Alternatively, synthetic antimicrobial peptides have been proposed as recognition agents, enabling detection and quantification of four bacterial strains, *Escherichia coli*, *Pseudomonas aeruginosa*, *Staphylococcus aureus*, and *Staphylococcus epidermidis* [108]. Synthetic antimicrobial peptides have advantages over immunological and DNA-based receptors that include low cost, large-scale production, and high stability. Moreover, they could also be used to inactivate pathogens [109,110]. Colorimetric biosensor strips fabricated with peptides immobilized on a gold chip were also reported for the detection of *Listeria monocytogenes* in milk and meat samples with an LOD of 2.17 × 10^2^ CFU/mL [111]. Recently, a fluorescent DNAzyme probe that specifically binds *E. coli* was developed and printed on a cyclo-olefin polymer transparent package (Figure 10) [112]. The study reported detection of *E. coli* in meat and apple-juice samples with a low LOD of 10^3^ CFU/mL.

Biosensors could be designed to incorporate materials with antimicrobial activity to create smart packaging [113]. Silver NPs (AgNPs), one of the most extensively studied antimicrobial agents, have been considered [114,115]. For example, AgNPs were imbedded into a hydroxypropyl methylcellulose polymer showing antimicrobial inhibition of *Escherichia coli* and *Staphylococcus aureus* [116]. In another study, acrylamide was copolymerized on paper with AgNPs entrapment, showing activity against *Escherichia coli* [117]. The use of AgNPs and other nanomaterials in active packaging applications is an active field of research. However the possible migration of NPs from the package to the foodstuff is a concern and should be considered in future research [118]. Natural antimicrobial materials, such as ethanol and essential-oil extracts, were suggested for possible use as emitters with protective functions in packaging [119]. Moreover, the potential to use the essential oils of oregano, rosemary, and garlic in edible films with antimicrobial activity has been demonstrated [120]. The idea of integrating biosensing platforms with systems for bacteria inactivation that can both detect and deactivate pathogens is emerging [10,121,122]. A 3D-ZnO nanorod-based electrochemical sensor was developed to both detect and kill bacteria showing the inactivation of 50% of bacteria [122]. Another example was reported using a surface-enhanced Raman scattering (SERS) silicon-wafer multifunctional chip [121]. The chip was modified with AgNPs to deactivate bacteria, and 4-mercaptophenylboronic acid to detect the pathogens. The assay measured *E. coli* and *S. aureus* down to 10^2^ cell/mL, with 60% deactivation efficiency at a bacterial concentration of 0.5–2 × 10^2^ CFU/mL. Most sensors were developed to demonstrate proof of concept. Issues regarding the safety and hazardous effect of antimicrobials (e.g., the release of Ag) need to be clarified before considering such platforms for food packaging. Moreover, reported sensors should be designed to meet regulatory limits and extensive validation should be provided.

### 4.3. Biosensors for Food Adulteration, Authenticity, and Toxicicity Assessment

Food adulteration is based on the change of food composition for the purpose of financial profit regardless safety consideration. Some examples including using illegal Sudan dyes in foods, bovine addition to chicken fillets [123], and the counterfeiting of olive oil [124]. Food adulteration is a growing concern, which creates the necessity for reliable analysis methods. A serious safety issue is adulteration of milk products with melamine due to its property to increase the apparent level of protein content as determined by protein quantification assay (Kjeldahl method). The incident resulted in about 300,000 sick children and six deaths [125]. An antibody-based optical biosensor was developed for melamine detection [126]. A polyclonal antibody was immobilized on the surface of an SPR biosensor, resulting in a sensitive platform measuring IC_50_ of 67.9 ng/mL. The sensor was selective for melamine but it showed cross reactivity with cyromazine, an insecticide that decays to form melamine. A label-free AgNP colorimetric sensor was developed to detect melamine in milk [127]. Yellow-red color was produced by AgNPs in presence of melamine as a result of aggregation. The method was able to detect down to 2.32 μM, lower than the Food and Drug Administration (FDA)-allowed limit of 20 µM [122]. Ni et al. (2014) [128] reported that melamine enhances the peroxidase-like activity of AuNPs in presence of 3,3′,5,5′-tetramethlybenzidine (TMB) and H_2_O_2_, generating a blue color formation. The method showed high sensitivity with an LOD of 0.2 nM.

Moreover, biosensors have been described to confirm the claimed composition and concentration of food products. Ethanol content has been estimated in alcoholic beverages by electrochemical biosensors based on immobilized alcohol oxidase enzyme [129]. An alcohol dehydrogenase-based biosensor was developed in presence of Meldola’s Blue on multiwall carbon nanotubes [130]. The linear range was 0.05–10 mM ethanol. Glucose evaluation in food products was also determined by an amperometric biosensor based on electropolymerized thin films [131] or based on carbon-nanotube osmium polymers [132].

The determination of polyphenol content in vegetable oils enables the estimation of the product’s antioxidant capacity. An HPR-based electrochemical biosensor was designed to evaluate chlorogenic acid content in vegetable oil. The sensor showed a sensitivity of 0.7 µM comparable with conventional detection methods [133]. An electrochemical biosensor was developed to discriminate between different types of vegetable oils (e.g., olive oil, sunflower oil, and corn oil), as well as the ability to differentiate between different types of olive oil (e.g., extra virgin, virgin, etc.) [134]. The working principle is based on the distinct polyphenol content, resulting in different electrochemical signals. Several types of sensors that utilize nanomaterials as a means to increase detection sensitivity and portability have been developed [135]. A portable paper-based assay was developed to determine the antioxidant capacity using ceria NP-modified paper. NPs interact with antioxidant compounds by means of surface and redox reaction, producing a colorimetric response in the range of 20–400 μM for several tested antioxidants, such as ascorbic acid, gallic acid, vanillic acid, quercetin, caffeic acid, and epigallocatechin gallate [136]. In other works, AgNPs were entrapped in poly (vinyl alcohol) (PVA) to develop a colorimetric sensor for quantifying antioxidant capacity, with an example demonstrated for the detection of gallic acid. The detection principle was based on seed-mediated NP growth. Ag^+^ was reduced to Ag^0^ that accumulated on the sensor surface, showing an increase on the particle surface. The PVA in the sensor acts as a catalyst for the reduction of Ag^+^ by gallic acid by providing AgNPs as nucleation seeds. Once the seed is formed, it accumulates on the PVA–AgNP surface leading to a red shift. The method provided a linear range from 25 to 200 μM and an LOD of 22.1 μM [137].

Other adulterants, such as monosodium glutamate (MGM) in food are also of interest. L-glutamate is a natural amino acid, and it is usually added to enhance food flavor and to add the umami taste to food. It has been reported that L-glutamate enhances food intake with no health risks [138]. However; it has been shown that L-glutamate has neuroexcitatory action [139]. Therefore, developing reliable methods to evaluate glutamate content in food products is important. An amperometric biosensor was developed by immobilizing glutamate oxidase on a Prussian Blue-modified electrode [140]. The biosensor was able to detect L-glutamate with an LOD of 0.7 nM. An electrochemical biosensor constructed by functionalization of a platinum anode with a thin film of Nafion and glutamate oxidase was able to selectively measure glutamate concentration as low as 0.3 µM [141].

The widespread use of pesticides in agriculture leads to their accumulation in soil, ground water, and crops. Due to their inherent toxicity, a control of pesticide levels in food products is necessary. Biosensors based on acetylcholinesterase (AChE) inhibition have been reported for the detection of carbamates and organophosphate used in insecticides [142,143,144,145]. Andreescu et al. (2002) [146] reported a comparative study of three AChE immobilization approaches on screen-printed electrodes by bioincapsulation in sol-gel composites, metal-chelate affinity, and entrapment in a photopolymerizable polymer. The enzyme entrapped in a polymer matrix showed stability for more than six months. The LOD for organophosphate-insecticide detection was in the range of 1–10 nM. As an alternative, biosensors based on organophosphorus hydrolase, which catalyzes the hydrolysis of organophosphorous pesticides, were also developed. Carbon black and mesoporous carbon-modified electrodes exhibited high sensitivity for the detection of p-nitrophenol resulted from hydrolase reaction of organophosphate [147]. The method was able to detect concentrations as low as 0.12 µM.

Another analyte of interest in the food sector is bisphenol A (BPA). BPA is a monomer used in the manufacture of polycarbonate and epoxy resin employed in the fabrication of a variety of food packaging such as water bottles, feeding bottles and coating material for processed food cans [148,149]. Exposure to BPA is associated with health risks and a ban on the use of BPA in children products is under debate since 2008 [150]. The FDA has banned the use of polycarbonates in baby bottles and spill-proof cups and the use of BPA-based coatings in packaging for infant milk in 2012 [151,152]. As a result, BPA detection is an emerging research topic in the sensing field. Alkasir et al. (2012) [153] developed an enzyme-based NP-functionalized electrochemical biosensor for quick and sensitive BPA detection. The biosensor modified with Ni NPs exhibited higher sensitivity and a lower LOD of 7.1 nM, in comparison with the biosensors modified with Fe_2_O_3_ (LOD, 8.3 nM) and AuNPs (LOD, 10 nM). Moreover, a paper-based colorimetric biosensor was developed for the detection of phenolic compounds, including BPA. The paper sensors showed an LOD of 0.86 µM and demonstrated high stability during 260 days, preserving 92% of its activity. BPA was estimated in drinking plastic water bottles and beverages cans using the method developed by Deng et al. (2014) [154]. The electrochemical biosensor employed a molecularly imprinted chitosan-graphene composite to selectively detect BPA with a reported LOD of 6.0 nM.

## 5. Conclusions and Future Trends

The implementation of chemical sensors, biological sensors, and indicator labels in smart and active packaging, as well as the development of methods enabling freshness investigation of food products and crops, is a growing field of study. While significant progress has been achieved, there is still a need to demonstrate the functionality of biosensing devices in realistic settings for evaluating the quality of packaged food. One of the main challenges in this field is the complexity of food samples and the difficulty to measure markers of degradation directly in closed packaging without prior sample treatment. Most successful examples of sensors to date are for the detection of volatile compounds, such as amines and ethylene. In the current development status, most food biosensors still need food-sample pretreatment. Future developments should be directed towards decreasing the detection limit and increasing the possibility to measure markers upon simple contact with the sample. Validation of measurements on large amounts of samples is also needed before any implementation in the field. In the biosensing field, there is a need to further simplify detection systems to avoid the use of multiple steps or reagents, decrease the cost, and miniaturize the sensor. Increasing the stability of the biological element in biosensing designs and ensuring long operability time during storage in packaged food is another challenge that needs to be addressed, especially for systems that incorporate sensitive biorecognition elements such as enzymes or antibodies. The effect of environmental parameters should also be established. Most sensors that have been reported in the scientific literature are not commercialized. These are in their vast majority in the initial design/proof of concept stage and require significant efforts to further develop into a marketable product to meet health concerns and current regulation. Additionally, integration of materials in smart packaging and food sensors should be done with full consideration of safety regulations due to the potential migration and contact with food.

Achieving multifunctionality through the combined use of detection and food-preservation methods is another growing field of research. Integration of these technologies is a challenge, but the rapid development of new antimicrobial agents and their assembly in functional coatings is expected to positively impact development. The use of nanomaterials in both sensing and packaging technologies is also growing and has demonstrated promising potential. However, toxicity concerns and safety need to be evaluated for promoting further use of nanotechnologies in the food industry. Moreover, ensuring connectivity of sensing devices and developing wireless, independently operated sensors are needed to facilitate the rapid monitoring of a large number of samples and to provide real-time status during shipping and long-term storage.
